# Supplementary material for: GraphATT-DTA: Attention-Based Novel Representation of Interaction to Predict Drug-Target Binding Affinity
Source: Biomedicines. 2022 Dec 27;11(1):67. doi: 10.3390/biomedicines11010067 (PMC9855982; doi:10.3390/biomedicines11010067)
Supplement: Supplementary file 1 [file biomedicines-11-00067-s001.zip › biomedicines-1972354-supplementary.pdf]

Supporting Information

GraphATT-DTA: Attention-Based Novel Representation of Interaction to Predict Drug-Target Binding Affinity

Haelee Bae [1], **Hojung Nam** [1,2,3,*]

[1] AI graduate school, Gwangju Institute of Science and Technology, 123 Cheomdangwagi-ro, Buk-gu, Gwangju, 61005, Republic of Korea
[2] School of Electrical Engineering and Computer Science, Gwangju Institute of Science and Technology, 123 Cheomdangwagi-ro, Buk-gu, Gwangju, 61005, Republic of Korea
[3] Center for AI-Applied High Efficiency Drug Discovery (AHEDD), Gwangju Institute of Science and Technology, 123 Cheomdangwagi-ro, Buk-gu, Gwangju 61005, Republic of Korea

*Correspondence: hjnam@gist.ac.kr

Training dataset and 5-fold cross-validation performance

Supplementary Table S1. Davis and BindingDB test dataset statistics

	Davis test	BindingDB	Duplicate
**Compound**	68	4,076	40
**Protein**	442	509	336
**Drug Target Pair**	5,010	14,505	551

Supplementary Table S2. Results of 5-fold cross-validation

	GCN	GAT	GIN	MPNN	DMPNN
Layer1	0.227	0.246	0.371	0.227	0.222
Layer2	0.223	0.255	0.223	0.221	0.225
Layer3	0.223	0.252	0.224	0.224	0.227
Layer4	0.223		0.232	0.218	0.225
Layer5	0.233		0.235	0.216	0.222

MSE during 5-fold cross-validation progress on Davis dataset

(a) GCN

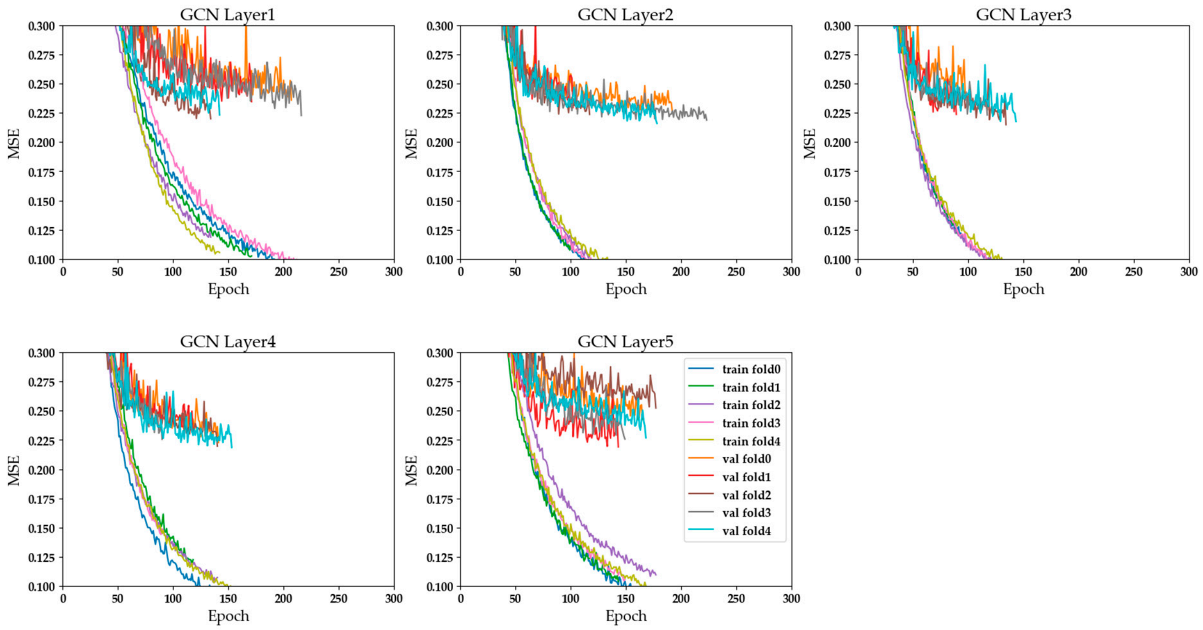

(b) GAT

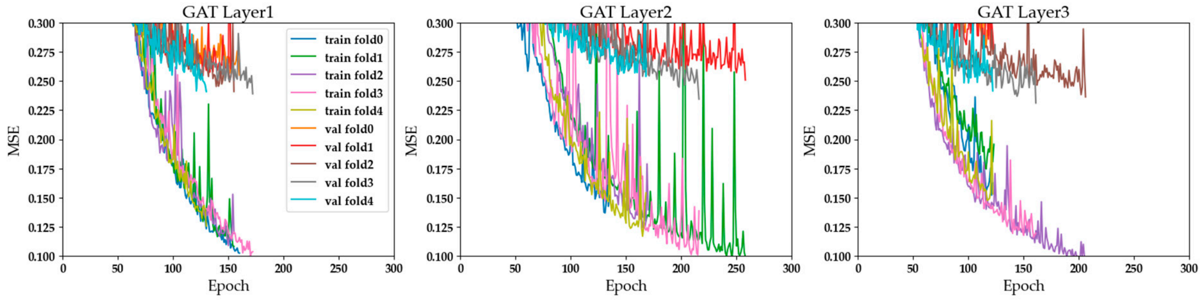

(c) GIN

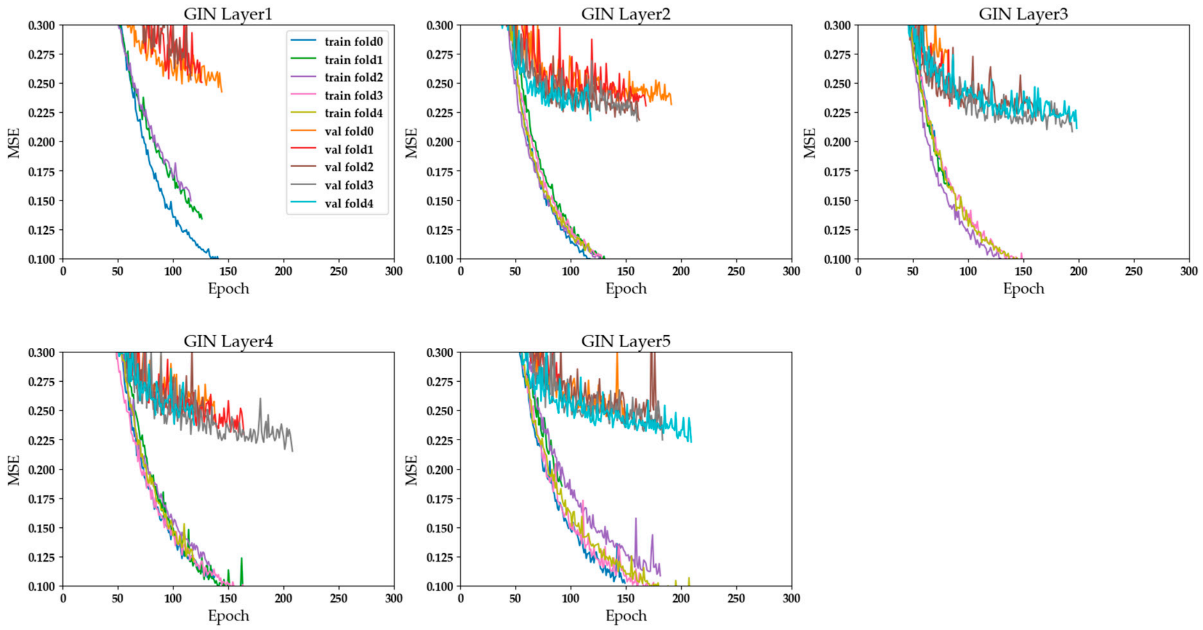
GIN Layer1
GIN Layer2
GIN Layer3
GIN Layer4
GIN Layer5
train fold0
train fold1
train fold2
train fold3
train fold4
val fold0
val fold1
val fold2
val fold3
val fold4
MSE
Epoch
0.300
0.275
0.250
0.225
0.200
0.175
0.150
0.125
0.100
0
50
100
150
200
250
300

(d) MPNN

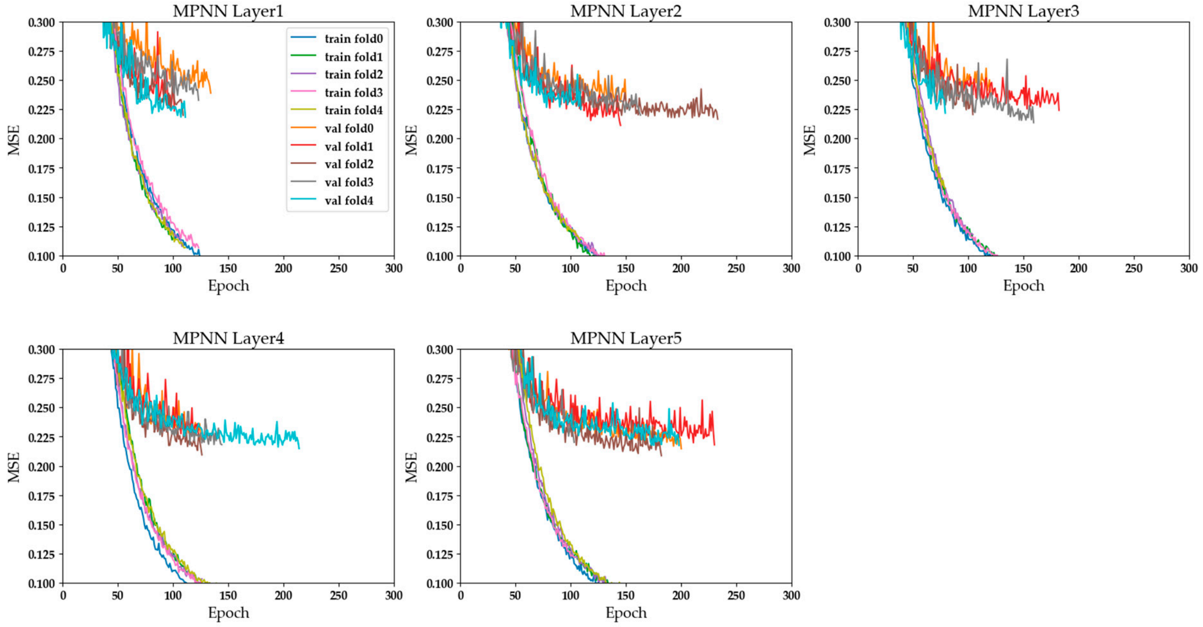
MPNN Layer1
MPNN Layer2
MPNN Layer3
MPNN Layer4
MPNN Layer5
train fold0
train fold1
train fold2
train fold3
train fold4
val fold0
val fold1
val fold2
val fold3
val fold4
MSE
Epoch
0.300
0.275
0.250
0.225
0.200
0.175
0.150
0.125
0.100
0
50
100
150
200
250
300

(e) DMPNN

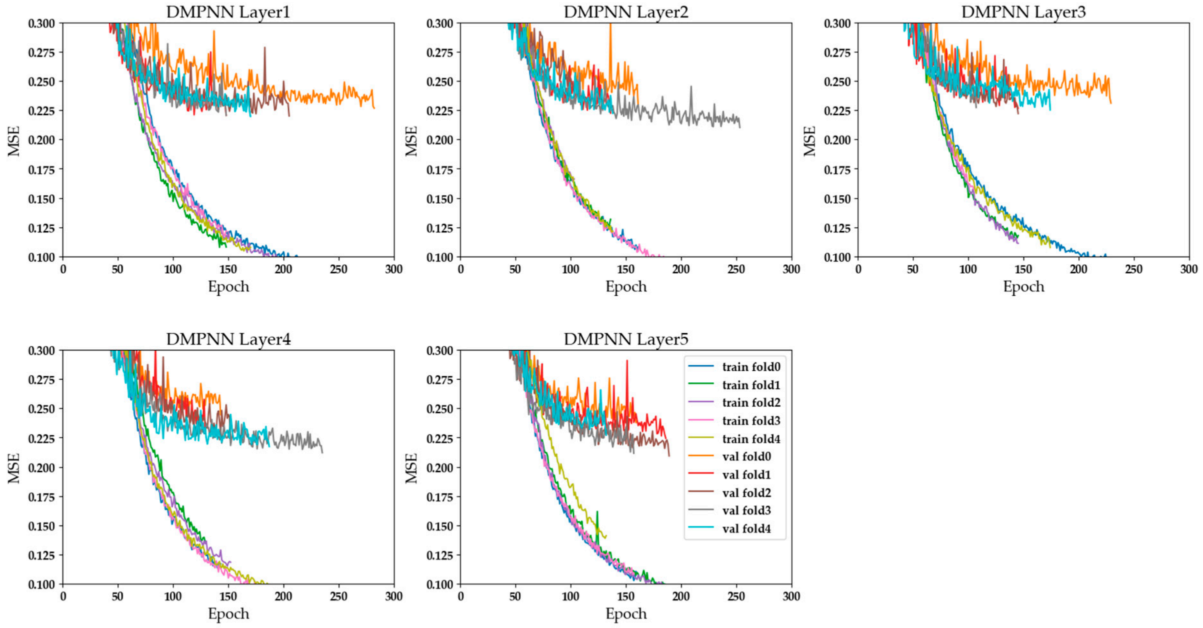

Supplementary Figure S1. MSE during 5-fold cross-validation progress on Davis dataset.

Prediction performance per compound scaffold

An additional analysis was conducted to demonstrate the DTA prediction performance per a drug class in terms of the unique chemical compound scaffold.

First, we applied the Bemis-Murcko scaffold splitting [36] to 68 compounds of Davis's test set to divide them into groups of a unique scaffold. As a result, the 68 compounds were categorized into 68 distinct scaffolds, confirming the diversity of compound structures of the test set in the Davis dataset.

Among the 68 scaffold classes, we found that many classes have a biased distribution of pKd values. A total of 53 classes is found to have pKd values that are near 5, the affinity value representing a compound's negative interaction with a protein. Thus, we filtered out classes with a ratio of negative DTA (pKd=5) greater than 50% to demonstrate the generalized prediction performance (Supplementary Figure S2).

Finally, fifteen scaffold classes remained, and we compared the DTA prediction performance for each class with high-performance baseline models, FusionDTA and ML-DTI.

As a result, we confirmed that different methods for determining DTA have overall similar prediction performance per a drug scaffold, indicating that no particular prediction model has a specially high or low prediction performance for a specific scaffold (Supplementary Figure S3). Interestingly, we observed that the scaffold of compound 10138260 has a high overall prediction performance, whereas the scaffold of compound 25243800 has a low overall prediction performance.

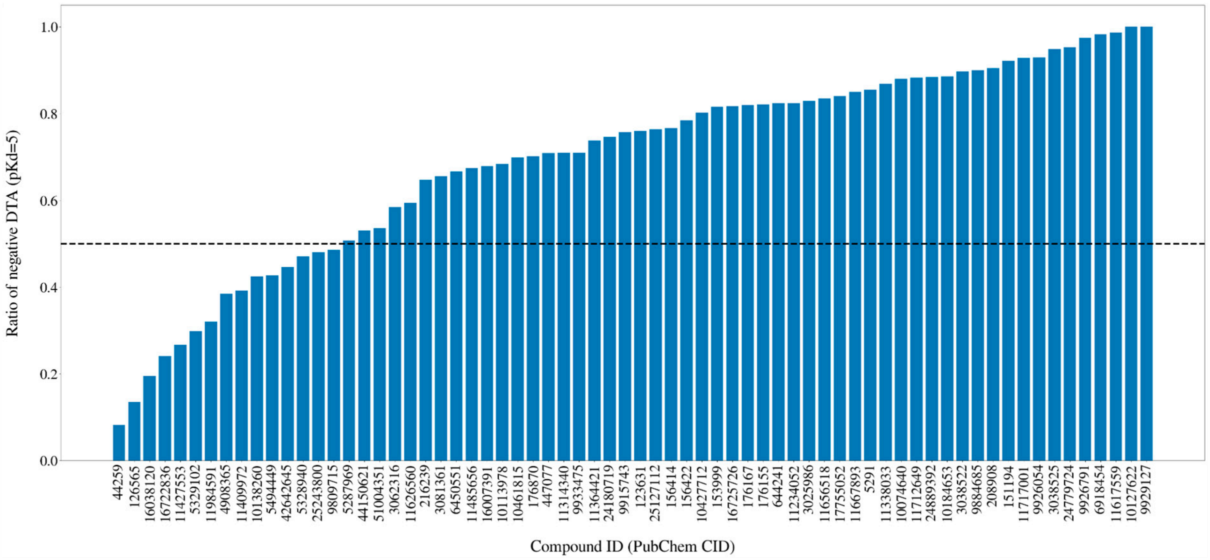

Supplementary Figure S2. The ratio of negative DTA for each compound. The distribution of pKd values for each compound is investigated. A total of 53 classes is found to have pKd values that are near 5, the affinity value representing a compound's negative interaction with a protein.

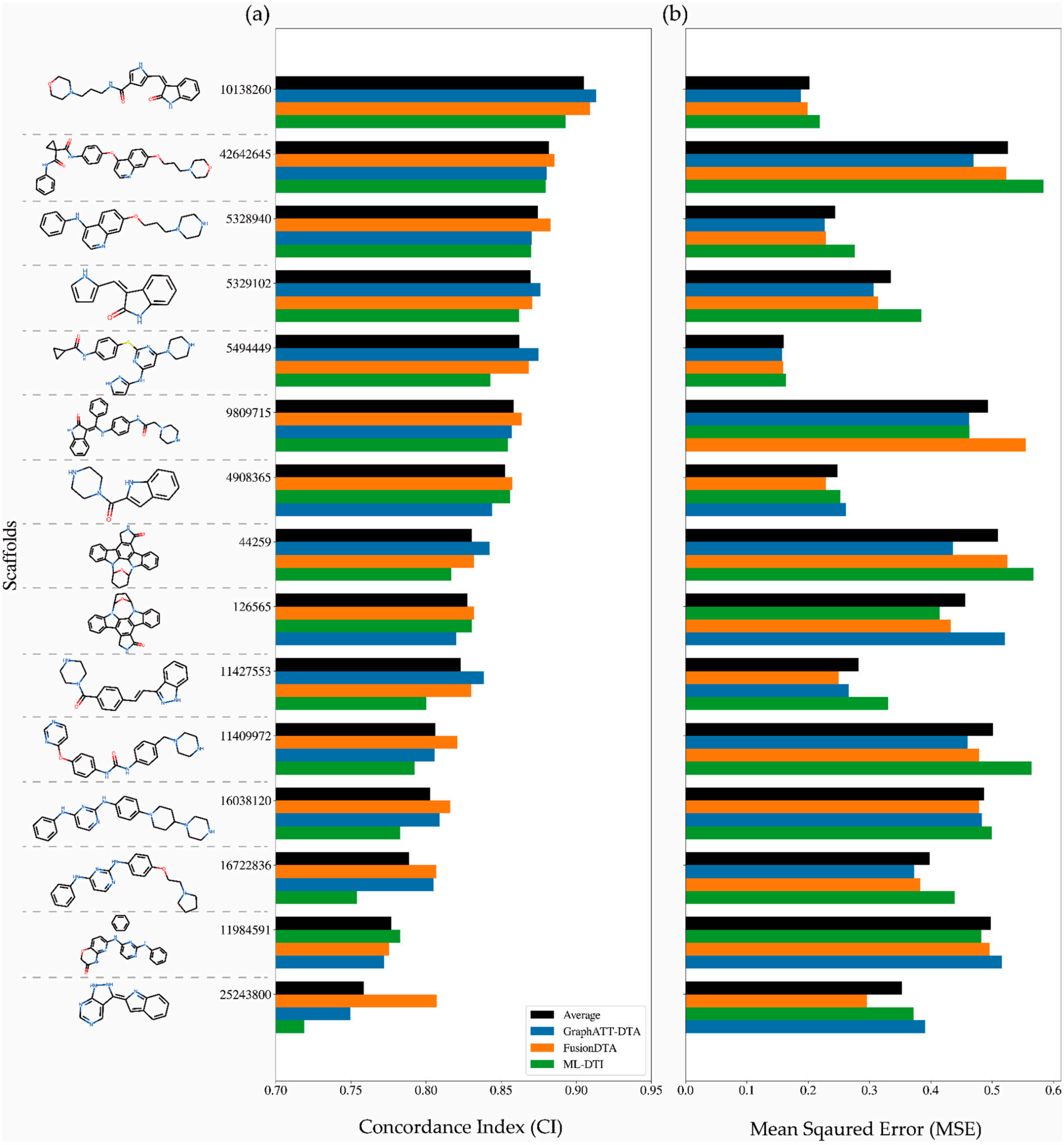

Supplementary Figure S3. Performance comparison of GraphATT-DTA and baseline models of each scaffold. (a) Concordance Index (CI) (b) Mean Squared Error (MSE).

Prediction from GraphATT-DTA model for BindingDB dataset

In general, the training data is a subset of real-world data. Therefore, if a machine learning model is continuously trained with training data, the error for the training data decreases, but the error increases for the real-world data. This is because the model learns the generic pattern of the training data instead of the general pattern of all data, and therefore fits the training data too closely. In this regard, it is crucial to validate the generalizability of the model using external data that can objectively evaluate the model's performance.

To construct the external dataset, we used BindingDB which has binding affinities between target proteins and compounds collected from literature, PubChem BioAssay, and ChEMBL. We extracted the human kinase protein-compound pairs that have Kd value. The data consists of 509 kinase proteins, 4,076 compounds, and 14,505 interactions. Although this is not all of the data from the real world, the composition of various compounds and kinase proteins reflects the data as closely as possible.

For the result external test with the BindingDB dataset, GraphATT-DTA achieved an MSE of 1.582 and a CI of 0.651. Local interaction modeling methods yielded superior results because the attention mechanism led to a more informative representation of the model. In preventing overfitting, early stopping also appears to have played an important role. In comparison to other models, GraphATT-DTA consistently demonstrated superior performance on the Davis and BindingDB datasets. However, there were differences in performance between the two. We hypothesize that disparities between the predicted affinity values and the actual affinity values (pKd) in the two datasets contributed to the performance decline. That is, in the Davis dataset, the lowest pKd value was five (10 μM). However, for the BindingDB dataset, many samples had values lower than five (Supplementary Figure S4).

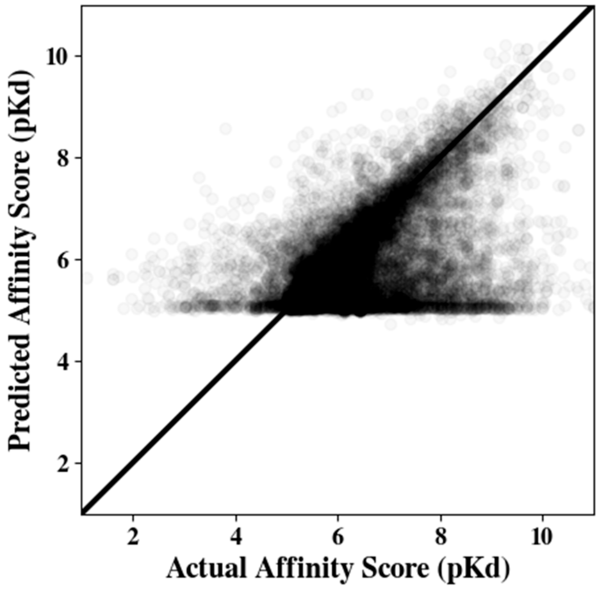

Supplementary Figure S4. Prediction of the GraphATT-DTA model on the BindingDB external testing dataset, and the scatterplot of predicted affinity values vs. actual affinity (pKd) values. The black line represents $y = x$. With the training dataset, the lowest pKd value was five; thus, the model predicted values less than five to be close to five.

Visualization with Case Studies

(a) (b)

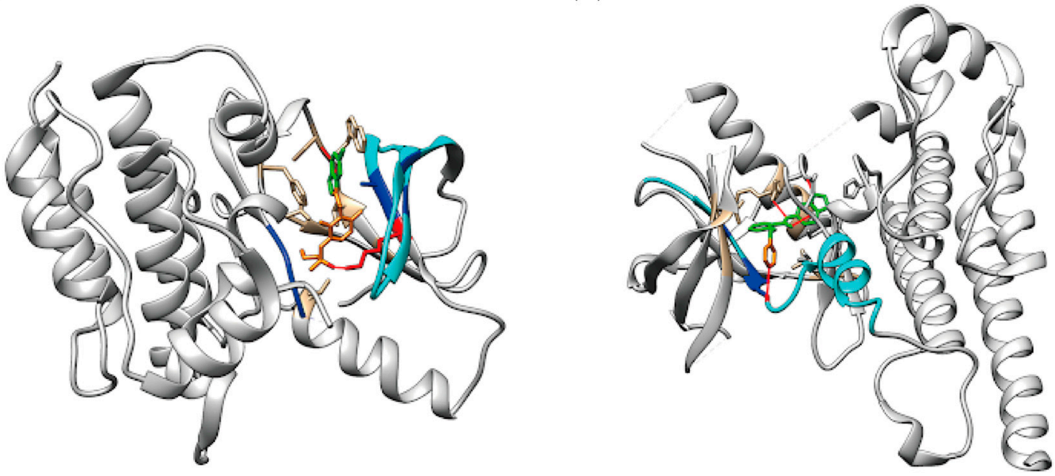

Supplementary Figure S5. Visualization of 3C4C and 3EFL complex. The binding affinity is predicted with GraphATT-DTA model and high attention regions are identified. (a) 3C4C is a complex of PLX4720 and serine/threonine-protein kinase B-Raf. (b)3EFL is a complex of motesanib and vascular endothelial growth factor receptor2 (VGFR2), the ligand is colored green, the ligand substructure where a high attention score as orange, protein regions where the true binding site with high attention score as dark blue, hydrogen bond and high attention score as red, not a binding site but predicted high attention score as light blue, binding site but not predicted high attention score as light yellow.

Simulation of compound structural modification

As GraphATT-DTA incorporates patterns of local-to-global interaction between a protein and a compound, we expect the model would be capable of capturing the effects of changes in local amino-acid residual changes of proteins or substructural changes of compounds on binding affinity. In this regard, we simulated the effects of compound structural modification on the impacts of binding affinity and identify the substructure that triggers decreasing the binding affinity.

In order to simulate the drug modification, we chose EGFR as the target protein and Canertinib (PubChem CID: 156414) as the target compound, which has the highest binding affinity for EGFR. The true binding affinity between EGFR and Canertinib is 9.721 pKd, and the predicted binding affinity by GraphATT-DTA is 9.415 pKd.

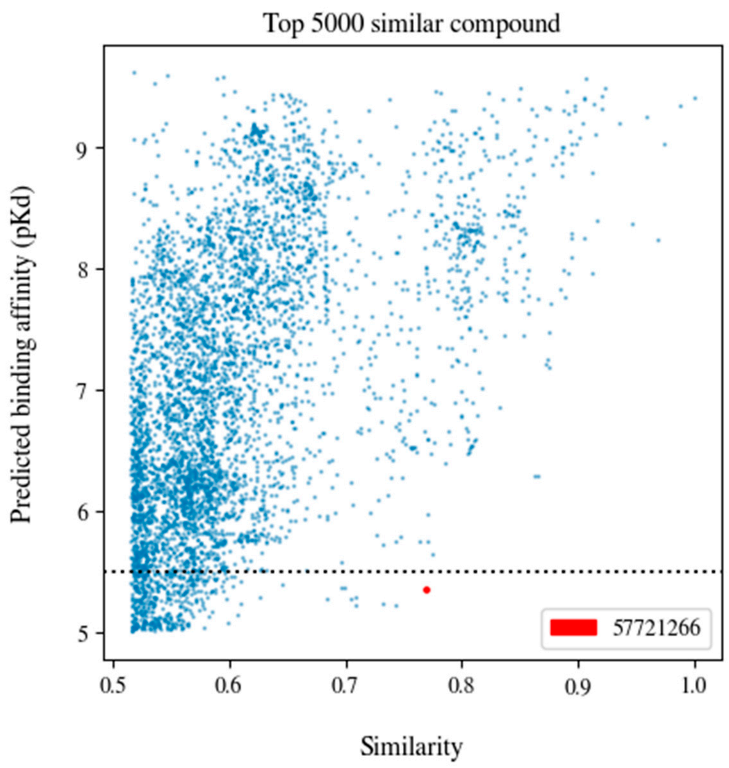

Supplementary Figure S6. Top 5000 similar compounds with Canertinib.

Next, in order to mimic the process of generating derivatives from a target compound without modifying its substructure, we selected 5,000 compounds from 1,584,910 ChEMBL compounds with the most similar structure to the target compound and predicted their pKd values (Supplementary Figure S6). Similarity with the target compound is plotted on the x-axis and predicted binding affinity is plotted on the y-axis for all 5000 compounds.

We set the pKd value 5.5 as a strict binding threshold to define the negative interaction between the target protein and the target compound [25,37]. The compound with the highest similarity to Canertinib that is predicted to have a negative interaction is PubChemCID57721266, which has a similarity score of 0.76 and a binding affinity prediction of 5.34. We identified the two substructures of Canertinib as the primary modifications (Supplementary Figure S7).

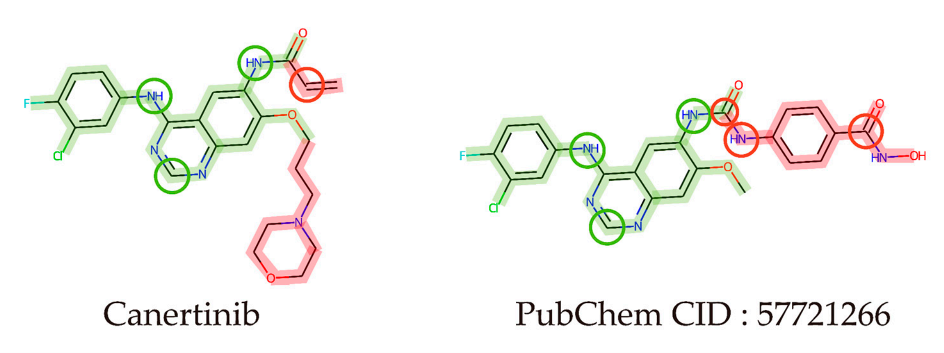

Supplementary Figure S7. The structure of Canertinib and 57721266 with high attention score. The green line indicates the shared substructures and the red line indicates the different substructures. The green circle indicates common high-score atoms and the red circle is the compounds' specific high-score atoms.

When we observed the attention analysis, we confirmed that Canertinib and PubChemCID57721266 commonly give high scores to N and C (green circle). Also, each compound gives high attention scores to its specific substructures (red circle). We can speculate that the compound's specific substructure with high attention scores in the PubChemCID57721266 compound may trigger a decrease in binding affinity.

Mutational effects on proteins

In order to observe the affinity prediction performance for mutated proteins, we collected mutant protein sequences and their binding affinity scores from BindingDB. We used the missense mutation protein sequence of FLT3 and EGFR and their interactions with 23 and 21 compounds, respectively, and investigated the changes between experimentally measured and predicted affinity values. However, no SOTA models have yet incorporated the concept of mutational alteration effects on protein sequence for modeling DTA. Consequently, the quantification of mutational effects on binding affinity remains limited. Thus, in this analysis, we evaluated whether our model could capture the changes in the directions of the affinity score rather than measuring the magnitude of affinity alteration.

The model correctly predicted the affinity increase/decrease direction in 13 of 23 cases for the FLT3 (N841I) mutant to the FLT3 gene (odd ratio: 1.3), 15 of 21 cases for the EGFR (G719C) mutant to the EGFR gene (odd ratio: 2.5) (Supplementary Table S3, S4).

In the FLT3 mutation (Supplementary Figure S8(a)), FusionDTA correctly predicts the 12 cases (odd ratio: 1.09) and ML-DTI predicts 7 cases (odd ratio: 0.438). In the EGFR mutation (Supplementary Figure S8(b)), FusionDTA correctly predicts the 8 cases (odd ratio: 0.615) and ML-DTI 8 cases (odd ratio: 0.667). Although GraphATT-DTA consistently performs well across the two mutations, there is a range disparity between the true and predicted pKd differences. For future research, it is necessary to develop a deep-learning model that can identify small-scale alterations in a protein sequence and apply their effects to predicting DTIs and DTAs.

Supplementary Table S3. FLT3 contingency table (odd ratio: 1.3).

Predicted \ Experimental	Increase	Decrease
Increase	3	7
Decrease	3	10

Supplementary Table S4. EGFR contingency table (odd ratio: 2.5).

Predicted \ Experimental	Increase	Decrease
Increase	6	3
Decrease	3	9

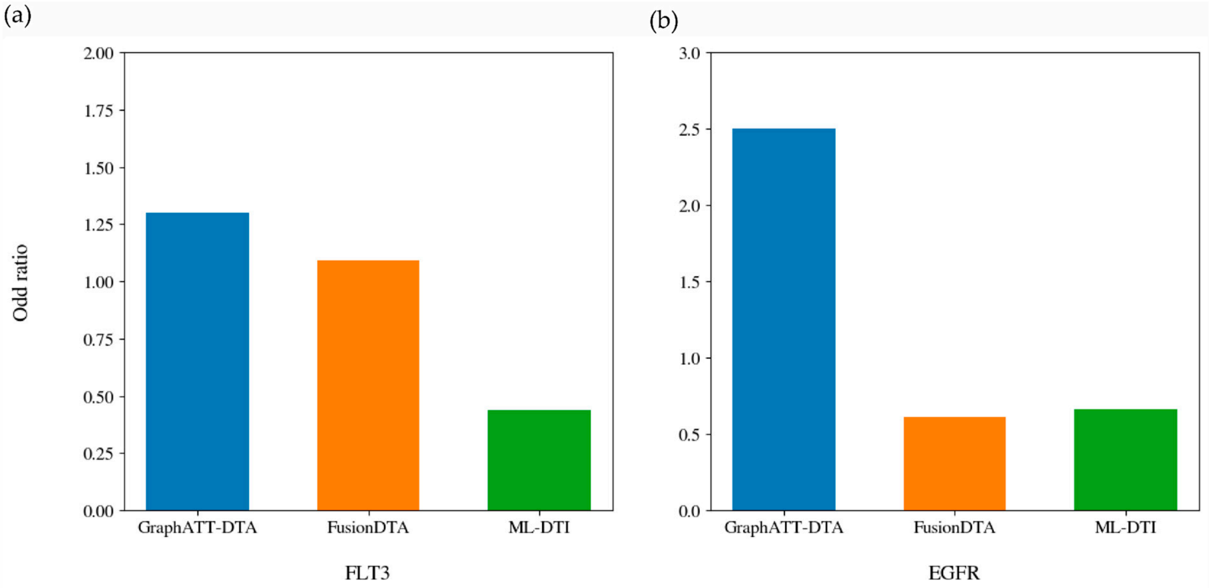

Supplementary Figure S8. Performance comparisons of GraphATT-DTA and baseline models on the difference between the affinity of wild and mutant proteins. (a)FLT3 (b) EGFR.

References

25. Davis, M.I.; Hunt, J.P.; Herrgard, S.; Ciceri, P.; Wodicka, L.M.; Pallares, G.; Hocker, M.; Treiber, D.K.; Zarrinkar, P.P. Comprehensive analysis of kinase inhibitor selectivity. *Nat. Biotechnol.* **2011**, *29*, 1046–1051.
36. Bemis, G.W.; Murcko, M.A. The properties of known drugs. 1. Molecular frameworks. Journal of medicinal chemistry 1996, 39, 2887-2893.
37. Karaman, M.W.; Herrgard, S.; Treiber, D.K.; Gallant, P.; Atteridge, C.E.; Campbell, B.T.; Chan, K.W.; Ciceri, P.; Davis, M.I.; Edeen, P.T. A quantitative analysis of kinase inhibitor selectivity. *Nature biotechnology* **2008**, *26*, 127-132.
